# Brain signature of food and alcohol stimuli processing: a comparative EEG study

**DOI:** 10.3389/fnhum.2026.1748993

**Published:** 2026-05-15

**Authors:** Sümeyye Kizilisik, Duygu Duman, Amel Zitouni, Salvatore Campanella, Ardalan Aarabi, Mbarka Akounach, Harold Mouras

**Affiliations:** 1 UR-UPJV 4559, Functional Neurosciences Laboratory, Health Research Universitary Center, Medecine UFR, Picardy Jules Verne University, Amiens, France; 2 Laboratoire de Psychologie Medicale et d’Addictologie, ULB Neuroscience Institute (UNI), CHU Brugmann-Universite Libre de Bruxelles (U.L.B.), Bruxelles, Belgium

**Keywords:** alcohol, EEG, food, incentives, neural oscillations

## Abstract

**Background:**

Food and alcohol cues are potent motivational stimuli that engage neural systems supporting reward and approach–avoidance behavior. While EEG studies have largely emphasized event-related potentials, less is known about how sustained oscillatory dynamics differentiate appetitive cues with distinct biological and learned significance.

**Methods:**

EEG activity was recorded in healthy adults during passive viewing of food-related, alcohol-related, and matched neutral images using a paradigm optimized for prolonged exposure. Spectral power was analyzed across canonical frequency bands with cluster-based permutation statistics. Individual differences in alcohol use were assessed using the Alcohol Use Disorders Identification Test (AUDIT). Additional psychometric assessments included measures of depression (BDI-II), trait anxiety (STAI-Trait), eating behavior (DEBQ), and nicotine dependence (Fagerström).

**Results:**

Food and alcohol cues elicited dissociable oscillatory responses. Food images were associated with increased delta-band power over posterior and central regions, whereas alcohol images induced decreased delta power and increased alpha-band activity over parieto-occipital areas. These effects were most pronounced in individuals with higher AUDIT scores, while lower-AUDIT scorers showed no consistent oscillatory modulation. Psychometric scores indicated non-clinical levels of depression and anxiety across participants.

**Conclusion:**

The findings indicate that food and alcohol cues engage partially distinct oscillatory dynamics during passive viewing. EEG spectral measures capture sustained, state-dependent aspects of motivational processing, highlighting differences between biologically grounded and learned reward cues.

## 1 Introduction

From the early foundations of ethology, “motivated behaviors” have been defined as actions supporting survival by driving the organism toward biologically relevant goals (Eibl-Eibesfeldt and Kramer, 1958). Such behaviors inherently require interaction with the environment and thus imply a close coupling between emotion and action at the neural level (Mogenson et al., 1980; Lang, 2014). In humans, visual cues with strong motivational value—particularly food and psychoactive substances such as alcohol—reliably activate reward-related circuits involved in attention, decision-making and inhibitory control (Volkow et al., 2011). Investigating how these cues are processed at the neural level can therefore deepen our understanding of the mechanisms contributing to maladaptive behaviors, including compulsive eating and excessive alcohol use.

Electroencephalography (EEG) provides a temporally precise window into the dynamics of neural activity during exposure to motivationally salient stimuli. Time-locked responses (event-related potentials, ERPs) and oscillatory activity in different frequency ranges have both been used to characterize the processing of appetitive cues. Regarding food stimuli, several reviews have summarized the ERP components robustly elicited during passive viewing (Becker et al., 2016; Blechert et al., 2010; Hanlon et al., 2012; Nijs et al., 2008; Schwab et al., 2017; Stockburger et al., 2008, 2009a,b; Versace et al., 2015). Numerous empirical studies have shown larger P300 and Late Positive Potential (LPP) amplitudes for food compared to non-food pictures (Becker et al., 2016; Blechert et al., 2010; Hanlon et al., 2012; Nijs et al., 2008; Schwab et al., 2017; Stockburger et al., 2008, 2009a,b; Versace et al., 2015; Hung et al., 2020), reflecting enhanced attention and motivational value.

Beyond reward-related processing, recent studies have also emphasized the role of inhibitory control mechanisms in the processing of food-related cues. In particular, research comparing high- and low-calorie food stimuli suggests that highly appetitive foods can place increased demands on cognitive control systems, reflecting a tension between automatic approach tendencies and regulatory processes. For example, experimental studies using Go/No-Go paradigms have shown that inhibitory control may differ as a function of food category and caloric value (Bianco et al., 2023). In addition, cerebellar neuromodulation studies suggest that food-related inhibitory control relies on distributed neural circuits extending beyond classical prefrontal control systems (Foti et al., 2025; Duck et al., 2025). Although caloric content was not manipulated in the present study, these findings highlight the broader interaction between motivational salience and cognitive control processes during food cue processing.

Similar electrophysiological reactivity has been observed for alcohol cues, although the literature has focused mainly on alcohol use disorders. Early findings reported changes in EEG signal complexity during alcohol cue exposure in social drinkers (Kim et al., 2023). More recent work has shown increased P300 amplitude to alcohol-related stimuli (Kang et al., 2022) and stronger ERP responses and microstates in regular non-dependent drinkers compared to occasional drinkers (Wang et al., 2025). Additional ERP studies further reported enhanced P3 amplitudes in social drinkers and alcohol-dependent individuals viewing alcohol pictures (Bartholow et al., 2007; Namkoong et al., 2004), and high reliability and acute sensitisation of alcohol-elicited ERP responses (Cofresí et al., 2022a,b). Together, ERP evidence shows that both alcohol and food cues evoke heightened neural engagement, although potentially through distinct motivational dynamics. Altered oscillatory brain dynamics during emotional processing have also been reported in young binge drinkers, further supporting the relevance of spectral EEG approaches for investigating alcohol-related motivational processing (Huang et al., 2018).

Beyond time-locked event-related potentials, spectral EEG analyses provide a complementary framework to investigate the sustained neural dynamics underlying motivational processing. Oscillatory activity in low-frequency bands, particularly delta and theta, has been associated with reward anticipation, motivational drive, and salience attribution, while alpha-band activity has been linked to attentional regulation and inhibitory control mechanisms (Kamarajan et al., 2012; Crane et al., 2023).

In the context of appetitive cues, alterations in delta–theta activity have been proposed as potential neural markers of motivational engagement and preoccupation, including states related to hunger, craving, or withdrawal. Recent studies using EEG spectral approaches have shown that physiological and motivational states such as hunger versus satiety, food preoccupation, or nicotine withdrawal can be decoded from oscillatory activity patterns, highlighting the sensitivity of spectral dynamics to internal state variables (Kalahasti et al., 2024; Kaya et al., 2025; Choi et al., 2025). Although this literature remains limited and methodologically heterogeneous, these findings suggest that oscillatory EEG measures may capture tonic and state-dependent aspects of motivation that are not fully reflected in transient ERP responses. Importantly, oscillatory signatures do not map one-to-one onto specific psychological constructs; rather, they provide a functional window into interacting processes such as motivational salience, attentional engagement, and regulatory control, whose expression may vary across stimulus categories and individual states.

A direct comparison between alcohol and food stimuli is theoretically important because both categories trigger approach-oriented motivation, yet differ in sensory modality, consumption dynamics and learned associations. Such a contrast can help determine whether their neural processing relies on shared oscillatory mechanisms or engages distinct motivational pathways.

The present study investigated EEG oscillatory activity during passive viewing of alcohol-related, food-related and neutral visual stimuli, with the aim of identifying category-specific spectral signatures associated with motivational processing. Based on previous work on incentive cue reactivity, we hypothesized that alcohol- and food-related stimuli would elicit distinct patterns of oscillatory activity, particularly in low-frequency bands associated with motivation and attentional engagement. Although posturographic and event-related potential (ERP) signals were recorded simultaneously as part of a broader experimental protocol, the present manuscript focuses exclusively on sustained EEG oscillatory dynamics. Postural correlates of food and alcohol cue processing, as well as early ERP responses and their relationship with approach–avoidance behavior, have been reported in separate publications based on the same dataset (Duman et al., 2025; Zitouni et al., 2025). Accordingly, spectral power differences between alcohol, food, and neutral conditions were examined using cluster-based permutation analyses.

## 2 Methods

### 2.1 Participants

Alcohol use and cue-reactivity were investigated using a sample size chosen to be comparable with previous EEG studies on appetitive cue processing in healthy adults, which typically include between 40 and 60 participants. The aim was to align with methodological standards in the field and to ensure stable spectral estimates and robust cluster-based permutation statistics, while remaining within realistic constraints of recruitment and recording time. No formal *a priori* power analysis was conducted, as reliable effect size estimates for spectral cue-reactivity in passive viewing paradigms were not available at the time of study planning. Anticipating potential exclusions related to technical issues or data quality (e.g., artifact contamination), a minimum of 60 participants was targeted.

A total of 65 participants (34 females, 31 males; mean age = 24.98 ± 7.21 years) contacted the research team to take part in the experimental procedure. Participants were recruited through flyers, newspaper advertisements, and posters. To encourage participation, each participant received a €50 gift card. Inclusion criteria were as follows: (i) being healthy; (ii) right-handedness; (iii) no history of psychiatric disorders and no use of psychiatric medication in the months preceding the experiment; and (iv) no alcohol consumption within 24 h prior to the experiment. All participants provided written informed consent prior to participation.

Data selection was conducted according to predefined objective criteria to ensure data reliability and analytical validity and followed a three-stage procedure: pre-experimental, experimental, and post-experimental. During the pre-experimental stage, three individuals were excluded after questionnaire screening because they did not meet the inclusion criteria. During the experimental stage, five participants were excluded due to technical issues encountered during the recording session. Following these exclusions, data from 57 participants remained.

In the post-experimental stage, data pre-processing was applied to further ensure data quality. Participants were excluded based on excessive trial rejection, defined as a number of rejected trials exceeding one standard deviation above the group mean. Based on this criterion, 9 participants were excluded from the analyses. Consequently, data from 48 participants (24 females, 24 males; mean age = 25.19 ± 7.40 years) were included in the final analyses. Given that previous EEG cue-reactivity studies typically report sample sizes ranging from 20 to 40 participants, the final sample size of 48 participants is considered statistically adequate.

Finally, a *post-hoc* power analysis confirmed that the final sample of 48 participants provided >80% power to detect medium effect sizes (Cohen’s *d* = 0.5) in within-subject contrasts of spectral EEG activity across alcohol, food, and neutral conditions. This supports the robustness of the analyses and indicates that the study was sufficiently powered to detect the hypothesized effects.

The study protocol was approved by the Comité d’Ethique pour les Recherches Non Interventionnelles (CERNI, Université de Picardie Jules Verne, Amiens, France) and was conducted in accordance with the Declaration of Helsinki (World Medical Association, 2013).

### 2.2 Stimuli selection

The images shown to the participants during the experiment were selected by considering four conditions: alcohol, neutral alcohol, food (appetitive), and neutral food. In addition to this, several key criteria were taken into account while selecting suitable images. Images were retained only if they depicted a single object without people, were presented on a neutral uniform background, and were matched for angle, luminosity, color balance, and resolution. The selected images were modified according to these standards to ensure consistency and quality. To ensure the suitability of alcohol, neutral alcohol, food, and neutral food stimuli, three reliable databases were used: CROCUFID (Cross-Cultural Food Visual Database; Toet et al., 2019), ABPS (Amsterdam Beverage Picture Set; Pronk et al., 2015) and Australian Beverage Picture Set (Onie et al., 2020).

CROCUFID is a comprehensive database containing food images from different cultures. This database is a valuable resource for researchers studying food preferences, eating habits, and cultural differences. In this database, which was created with a standard photography protocol, all foods were photographed on a fixed background (white plate and fixed background), at a fixed angle (45°) with high resolution (1037 × 691 pixels). In order to provide sufficient variety in valence and arousal levels, images of fresh, foreign, moldy/spoiled, or partially consumed foods were also added. CROCUFID was used in the selection of neutral and appetitive food stimuli due to its features such as fixed background, realistic images, presentation of foods on a plate, presence of shadows, variety, and high resolution.

ABPS is a database focused on visual presentation of beverages and is widely used for studies investigating the psychological effects of alcohol consumption, thirst perception, and beverage preferences. The database contains 44 alcoholic and 33 non-alcoholic beverage images. Alcoholic beverage images include beer, wine, hard liquor, and alcopops, while non-alcoholic beverages include water, soda, and various other drinks. Object angle and lighting were controlled in the images. The size of the images is 500 × 500 pixels. The resolution enables analysis of packaging designs and beverage contents. ABPS also includes different presentation formats such as glass, bottle, or can.

Lastly, the Australian Beverage Picture Set was employed. This database covers a wide range of beverages including water, fruit juices, soft drinks, and alcoholic beverages. The images were carefully captured with a standardized background and uniform lighting to minimize visual variability across the research environment. The high-resolution images of beverages, both packaged and unpackaged, provide a resource for research examining consumer behavior, health effects associated with beverage choices, and perception of beverages.

A uniform white background was used in all images to ensure that participants focused only on the stimuli and to minimize distraction from background elements. Images that were natural and realistic were chosen. Images that did not fulfill these requirements were removed because artificial images that lack shadows or do not seem to be placed on a surface may produce unnatural effects on visual perception. Finally, databases containing high-resolution images were preferred to avoid pixelation. Additionally, gray-scale corrections were made to images with overly bright white backgrounds to ensure visual consistency.

Another important element in the selection of stimuli was the definition of neutral food and neutral alcohol stimuli. Neutral foods were considered to be plain foods without any particular color, taste or presentation. Examples included fruits and vegetables without any garnish or sauce (broccoli, zucchini, boiled vegetables without spices), plain cereals (rice, oats, quinoa), unsweetened porridge, simple protein sources such as hard-boiled eggs, unsweetened bread and whole grains, plain soups without any obvious ingredients or sauces, tofu, plain pasta, boiled potatoes and unsweetened yogurt Toet et al. (2018). Neutral alcohol stimuli, on the other hand, were defined as soft drinks.

A total of 208 images—52 for each of the four conditions—were selected from the identified databases and organized according to the given criteria. The complete list of stimuli included in each experimental condition is provided in Supplementary Table S1.

### 2.3 Experimental paradigm

The experimental paradigm developed by Luther et al. (2023) was used in this study. In this procedure, EEG and posturography measurements were integrated and recorded simultaneously. The present report examines the effects of visual stimuli (pleasant/unpleasant) on brain activity (EEG).

**Figure 1:**
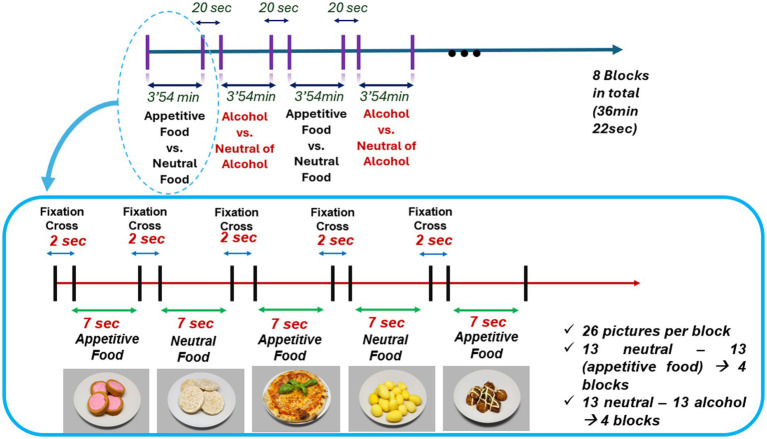
Overview of the experimental paradigm and illustration of one block.

Based on this design, a new experimental procedure was established. The paradigm was created using E-Prime software, and its visual layout is presented in Figure 1. The paradigm consists of 208 images divided into 8 blocks. Each block contains 26 images: 13 neutral and 13 either food- or alcohol-related stimuli. Blocks containing food stimuli were alternated with blocks containing alcohol stimuli. In total, 4 blocks present food-related images and 4 blocks present alcohol-related images. A 20-s rest period was introduced between each block, with an extended 3-min rest between the 4th and 5th blocks. Within each block, stimuli were presented for 7 s, followed by a 2-s fixation cross. The image order within each block was randomized. One example block of the paradigm is shown in Figure 1.

The stimulus duration (7 s) was selected based on previous paradigms combining EEG and posturography, in which prolonged visual exposure is required to reliably capture postural adjustments while preserving the quality of concurrent neural recordings (Luther et al., 2023). This timing represents the most compatible compromise for jointly investigating neural and postural dynamics within the same experimental protocol.

The 52 food, 52 neutral food, 52 alcohol, and 52 neutral alcohol images used in the paradigm were selected from validated databases, totaling 208 images presented to each participant during the experiment. Unlike Luther et al.’s (2023) original paradigm, each image was shown for 7 s instead of 2 s, as the shorter duration may be insufficient to detect posturographic changes in response to the stimuli. Longer exposure times are commonly used in posturography studies (Akounach et al., 2022; Mouras et al., 2015). Considering all segments and rest periods, the total duration of the experimental session was approximately 35 min.

### 2.4 Experimental procedure

The first phase of the study, the pre-experimental stage, began when participants were invited to the laboratory. After receiving detailed information about the experiment, an informed consent form prepared in two copies was signed by both the participant and the researcher conducting the study. One copy of the consent form was given to the participant.

To ensure participant anonymity, each individual was assigned a unique seven-character alphanumeric code. Throughout the study, no personal identifiers were used; all data were processed using these codes only. After obtaining the required signatures and assigning participant codes, a series of questionnaires were administered. Prior to completing the other questionnaires, participants underwent the Mini-Mental State Examination (MMSE), which was administered by a different researcher. Following the MMSE, participants completed a series of psychometric questionnaires in written form, as described in Section 2.5.1, and were instructed to respond to each item carefully and thoroughly. After the questionnaires were completed, the study proceeded to the experimental stage. Participants were taken to the experimental room where EEG and posturography recordings were performed simultaneously. Visual stimuli were presented according to the experimental paradigm described in Section 2.3.

After the approximately 35-min experimental session, participants were given time to rest. The post-experimental stage followed this rest period. Participants were asked to rate the images they had seen during the experiment on a scale from 1 to 9, based on several criteria: pleasantness, unpleasantness, consumption desire, approach desire, avoidance desire, and intensity. Once all sessions were completed, participants received a gift card and were asked to sign a document confirming its receipt.

### 2.5 Measures

#### 2.5.1 Psychometric data

Prior to the experimental procedure, participants completed a series of questionnaires designed to assess various psychological and behavioral characteristics. These questionnaires collected sociodemographic information, including dominant hand, physical activity level, and the presence of any diseases or disorders, while also incorporating validated measures to evaluate psychological and cognitive functions. In this study, the French versions of seven widely used and scientifically validated questionnaires were administered.

Participants’ depression levels were assessed using the Beck Depression Inventory-II (BDI-II), a widely used 21-item self-report questionnaire developed by Beck et al. (1996) to measure depressive symptoms and their severity (0–13: No depression, 14–19: Mild depression, 20–28: Moderate depression, 29–63: Severe depression). The State–Trait Anxiety Inventory (STAI-TRAIT), a 20-item anxiety assessment scale developed by Spielberger et al. (1971), was used to measure participants’ anxiety levels. The participant is asked to score these items from 1 (almost never) to 4 (almost always). Depressive symptoms (BDI-II) and anxiety levels (STAI-Trait) were assessed to characterize the sample rather than to define inclusion criteria. Mean BDI-II scores fell within the non-clinical range for both groups [see also Zitouni et al. (2025)], in line with the absence of diagnosed psychiatric disorders required for participation. Although a small number of participants displayed scores in the mild range, none reached the threshold corresponding to moderate or severe depression. These participants were retained because their removal did not alter the sample characteristics nor any of the behavioral or electrophysiological results. A similar pattern was observed for anxiety scores measured via the STAI-Trait, with all participants falling within the non-clinical range and no effect of anxiety on the dependent variables.

The Mini-Mental State Examination (MMSE), a widely used 30-item test designed to assess cognitive functions such as thinking, communication, comprehension, and memory, was administered to participants in an interactive manner. The test, developed by Kurlowicz and Wallace (1999), consists of several sections, including Orientation, Learning, Attention and Calculation, Recall, Language, and Constructive Praxis. Participants responded to the examiner’s questions in each section, and their cognitive health was evaluated based on their responses. Participants’ hand dominance was determined using the Edinburgh Handedness Inventory (Oldfield, 1971). It is a 10-item questionnaire designed to assess an individual’s dominant hand. For each item, participants indicate which hand they use while performing a specific activity. Another questionnaire, the Dutch Eating Behavior Questionnaire (DEBQ), developed by Van Strien et al. (1986), was administered to assess eating behaviors. This 33-item questionnaire evaluates three different eating behaviors in adults: emotional eating, external eating, and restrained eating. The participant’s alcohol consumption level is a crucial aspect of our study. Therefore, the Alcohol Use Disorders Identification Test (AUDIT) (Williams, 2014) was administered, a 10-item questionnaire designed to diagnose risky alcohol consumption or alcohol use disorder. Participants rated almost all questions, except for Questions 9 and 10, on a scale from 0 (never) to 4. For these two questions, they selected one of three options: 0, 2, or 4. To distinguish between levels of alcohol use, participants were divided into two groups—low and high alcohol consumers—based on a median split of their total AUDIT scores. Finally, the Fagerström Test (Heatherton et al., 1991) was used to assess tobacco/nicotine addiction. Participants responded to six questions, with scores categorized as follows: 0–2 (no addiction), 3–4 (low addiction), 5–6 (moderate addiction), 7–8 (strong addiction), and 9–10 (very strong addiction). The Fagerström Test was included to characterize potential individual differences related to nicotine use, which may influence reward sensitivity and cue-reactivity processes. In the present sample, nicotine dependence levels were generally low and were not associated with the behavioral or electrophysiological measures. Consequently, these variables were not included in the main statistical analyses.

#### 2.5.2 EEG data

EEG data were recorded using a BioSemi ActiveTwo system with a sampling frequency of 2048 Hz. The EEG setup consisted of a 64-channel BioSemi EEG cap with active electrodes arranged according to the international 10–20 system, connected to a 256-channel BioSemi ActiveTwo AD box. Pin-type electrodes with sintered Ag–AgCl tips were used. During acquisition, CMS and DRL electrodes served as reference and ground within the BioSemi active feedback loop. EEG data were subsequently re-referenced to the common average prior to preprocessing.

Posturography data were recorded simultaneously. At this stage, the durations of the stimuli sent to both systems (EEG and posturography) were checked and the parallel port was preferred as the option closest to synchronization. One parallel port transmitted the stimuli to the BIOPAC MP150 system. The other was connected to the Neurospec interface, which enabled precise synchronization between stimulus presentation and EEG recording.

#### 2.5.3 Rating data

Following the completion of posturographic data recording, participants proceeded to the subjective evaluation phase, where they assessed visual stimuli presented under predefined experimental conditions. This phase aimed to measure the psychological and emotional responses elicited by each image, facilitating the investigation of the relationship between posturographic data and subjective evaluations.

The evaluation phase used the same set of stimuli and experimental structure as described in Section 2.3. Each stimulus was displayed for 3 s, followed by a sequence of six standardized questions assessing Pleasantness, Unpleasantness, Approach Desire, Consumption Desire, Avoidance Desire, and Intensity on a 9-point scale.

The evaluation phase followed the same eight-block structure as the experimental phase, with 26 images per session and three-minute rest intervals between blocks.

Because the study was exploratory regarding the affective and motivational dimensions of cue-reactivity, the six rating scales (pleasantness, unpleasantness, approach desire, consumption desire, avoidance desire, and intensity) were analyzed separately. No strong *a priori* hypotheses were formulated for scale-specific effects, and therefore these analyses should be considered descriptive rather than confirmatory. The results were interpreted in terms of the global pattern across stimulus categories rather than in terms of scale-specific contrasts.

### 2.6 Data analyses

#### 2.6.1 EEG data

The present study specifically focused on frequency-domain EEG analyses in order to characterize sustained oscillatory activity during passive viewing of motivational stimuli. Accordingly, EEG analyses were restricted to spectral power measures, and event-related potential analyses were not included in this manuscript.

##### 2.6.1.1 Preprocessing

Each participant’s EEG data, recorded with the BioSemi system, were preprocessed in MATLAB with the FieldTrip toolbox (Oostenveld et al., 2011). Continuous EEG data were segmented into 7.5-s epochs, starting 0.5 s before stimulus onset and continuing for 7 s after the stimulus. A band-pass filter was applied between 1 and 100 Hz, and power line noise was removed using a notch filter at 50 Hz. All data were resampled to 512 Hz. Bad channels were identified and removed through visual inspection based on *z*-score, kurtosis, and variance. Artifacts such as eye blinks were detected using independent component analysis (ICA) and manually selected components were removed. Following ICA correction, neighborhood-based channel interpolation and average re-referencing were applied. After a second visual inspection, all remaining trials containing artifacts were rejected using FieldTrip procedures.

The resulting clean dataset was split into four experimental conditions for subsequent analysis.

In order to enhance data quality, the method proposed by Luther et al. (2023) was used as a reference. Based on this approach, 9 participants were completely excluded from the analysis. The number of rejected trials for each participant was assessed, and the mean and standard deviation of these values were calculated to establish a threshold. This threshold was defined as one standard deviation above the mean (mean + std). Participants whose number of rejected trials exceeded this threshold were excluded from the study to preserve the overall quality of the analysis and minimize the impact of artifact-contaminated signals.

This procedure resulted in the inclusion of 48 participants in the subsequent analyses.

##### 2.6.1.2 Power spectral analysis

Because the study was exploratory regarding the spectral correlates of cue-reactivity, power analyses were conducted separately for each canonical frequency band (delta, theta, alpha, beta). We acknowledge that this approach may increase the number of statistical comparisons; however, the use of cluster-based permutation tests strongly limits the risk of Type I error inflation.

Spectral analysis methods are applied to determine the distribution of the power of signals in frequency space and to interpret their characteristics, which shows how a stationary random and finite-length signal is distributed over the frequency band. Spectral analysis aims to reveal repeated or hidden behaviors in the signal and provides a methodological framework for estimating frequency-domain properties of EEG activity (Babadi and Brown, 2014; Zhang, 2019).

Power spectral density describes the power distribution of a signal over the frequency range (Sharma and Meena, 2024).

For spectral analysis, the entire 7-s stimulus period was selected for each condition. Power spectral density (PSD) was calculated using FieldTrip toolbox. Spectral power was obtained using the Hanning window in the 1–100 Hz frequency range in all EEG channels. This analysis was performed separately for all four conditions. Statistical comparisons regarding the EEG spectral power were performed over five classical frequency bands: delta (1–4 Hz), theta (4–8 Hz), alpha (8–12 Hz), beta (12–30 Hz) and gamma (30–100 Hz).

##### 2.6.1.3 Cluster-based permutation test (CBPT)

The problem of multiple comparisons has been a significant disadvantage in the statistical analysis of EEG data. This issue arises because the effect under investigation is tested across a large number of sensor–time pairs. The large number of statistical comparisons increases the risk of Type I errors, making it difficult to control the family-wise error rate. CBPT, which is a non-parametric statistical test, has overcome the limitations of classical parametric tests that can be used in this case and has become a reliable test that takes into account spatial (channels), temporal and frequency neighborhood relationships (Maris and Oostenveld, 2007).

This method is preferred to prevent false positive results and obtain more reliable findings, especially in multidimensional data such as EEG. It not only increases statistical power but also enables clearer visualization of the spatial and spectral locations of the observed effects.

The analyses were performed using the FieldTrip toolbox and the Monte Carlo method was applied with 5,000 permutations for statistical significance. In order to control the possibility of cumulative error, a cluster-level correction was made for multiple comparisons, and the threshold significance was determined as *p* < 0.05.

The analyses were conducted separately in five frequency bands. In each frequency band, the differences on the power spectra were visualized topographically and the statistically significant clusters were reported.

#### 2.6.2 Ratings data

All statistical analyses were conducted using R software (R Core Team, 2024). For each of the six subjective evaluation dimensions (pleasant, unpleasant, approach, avoidance, consumption, and intensity), four experimental conditions were compared: alcohol, neutral alcohol, food, and neutral food.

In order to assess the suitability of the data for parametric analysis, the Shapiro–Wilk normality test was applied to the rating values for each experimental condition. The Shapiro–Wilk test is a widely used and reliable method to test the normality of data distribution, especially in small and medium-sized samples. According to the *p* value obtained, results above 0.05 indicated that the data were normally distributed, while results below 0.05 indicated that the data were not normally distributed. Groups with sample sizes outside this range were excluded from the analysis. In addition, histograms and QQ plots were created for each variable and the distribution properties were evaluated visually.

Due to the non-normal distribution of the data assessed by visual inspection and confirmed by the Shapiro–Wilk normality test, the non-parametric Wilcoxon signed-rank test was used to compare the paired conditions.

To control for the risk of Type I error resulting from multiple comparisons, *p*-values were adjusted using the False Discovery Rate (FDR) correction method. A significance threshold of *q* < 0.05 was applied.

Data for each dimension were visualized using box plots and statistically significant differences were annotated directly in the graphs using asterisks.

#### 2.6.3 Psychometric modulation analyses

To investigate whether individual differences modulate neural reactivity associated with cues, additional exploratory analyses were conducted between questionnaire measures and condition-specific spectral reactivity. Reactivity indices were calculated as the power difference between the cue condition and the corresponding neutral condition (alcohol − neutral alcohol; food − neutral food) in frequency bands showing a significant cluster-level effect.

Relationships between these reactivity indices and questionnaire scores (AUDIT, BDI, STAI, and DEBQ subscales) were analyzed using Spearman rank correlation. To control for multiple comparisons, false discovery rate (FDR) correction was applied within each cue category. To evaluate the independence of significant relationships in more detail, partial correlation analyses were performed, controlling for depressive and anxiety symptom scores where appropriate.

## 3 Results

### 3.1 Psychometric data

The results of the psychometric tests are summarized in Table 1. The scores obtained from the BDI-II and STAI-Trait scales generally fall within the non-clinical range, indicating that there are no clinical levels of depression or anxiety symptoms in the sample. Alcohol use was assessed using the Alcohol Use Disorders Identification Test (AUDIT). Scores in our non-clinical sample mainly fell within the low-to-moderate range. To explore whether *relative* differences in alcohol use within this range were associated with behavioral and neural responses, we stratified participants into two subgroups based on the median of the AUDIT distribution: *lower-AUDIT scorers* and *higher-AUDIT scorers*. This approach is *data-driven and exploratory* and does not correspond to the clinical cut-off score of 8 used to identify hazardous drinking. Therefore, these subgroups reflect inter-individual variability in alcohol use within a healthy population, rather than clinically meaningful categories. Participants were divided into two groups based on a median score of 3. The low alcohol consumption group included 23 participants, while the high consumption group included 25 participants.

**Table 1:** Psychometric characteristics of the participants (*N* = 48).

Psychometric test	Mean ± SD	Range
BDI-II	9.65 ± 7.64	0–25
STAI-Trait	39.08 ± 9.96	20–62
MMSE	30 ± 0	30–30
DEBQ–emotional eating	2.17 ± 0.79	1–3.92
DEBQ–external eating	3.04 ± 0.63	1.9–4.4
DEBQ–restrained eating	2.18 ± 0.87	1–4.9
AUDIT	6.13 ± 6.29	0–24
Fagerström	0.44 ± 1.38	0–6

### 3.2 Subjective ratings

Given the number of rating variables and associated statistical tests, the results below should be interpreted with caution. The aim of these analyses was to characterize the overall subjective pattern of cue-reactivity across the three stimulus categories rather than to establish statistically independent effects for each scale.

#### 3.2.1 Globally

**Figure 2:**
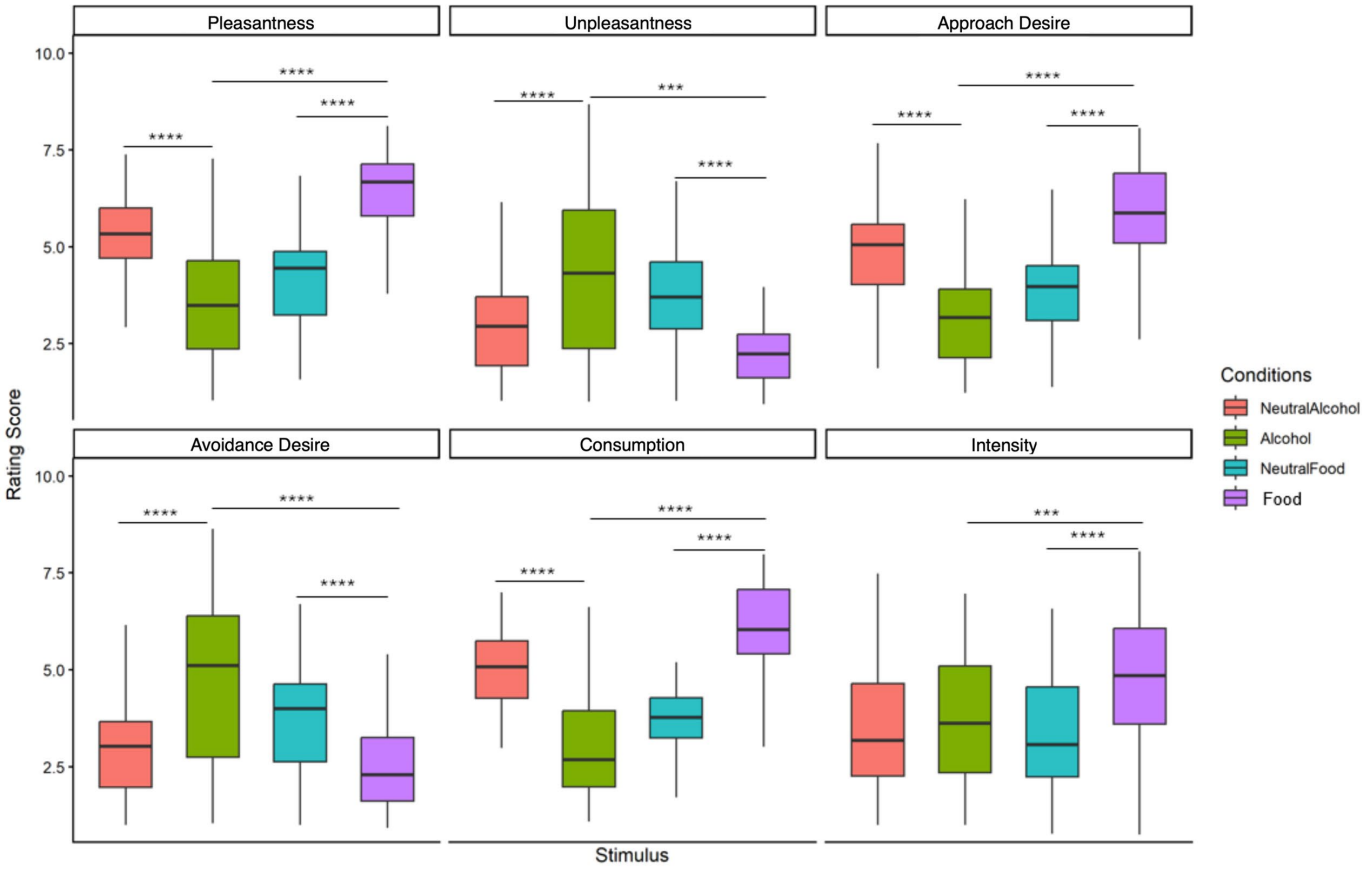
Global analysis of rating scores across four stimulus conditions for six emotional and motivational dimensions. Ratings were provided on a 9-point scale. Statistical differences were assessed using the Wilcoxon signed-rank test (^***^p < 0.001, ^****^p < 0.0001).

Significant differences were observed across most dimensions. Comparisons between alcohol and neutral alcohol stimuli were significant for the pleasant, unpleasant, approach, avoidance, and consumption dimensions (all *p* < 0.001), whereas the Intensity dimension was not significant (*p* > 0.05). Comparisons between appetitive food and neutral food stimuli revealed significant differences across all dimensions (all *p* < 0.0001). Notably, appetitive food stimuli consistently elicited the highest ratings across several motivational dimensions, including pleasantness, approach desire, and consumption desire, clearly differentiating them from both alcohol-related and neutral stimuli (see Figure 2).

#### 3.2.2 Lower-AUDIT scorers

**Figure 3:**
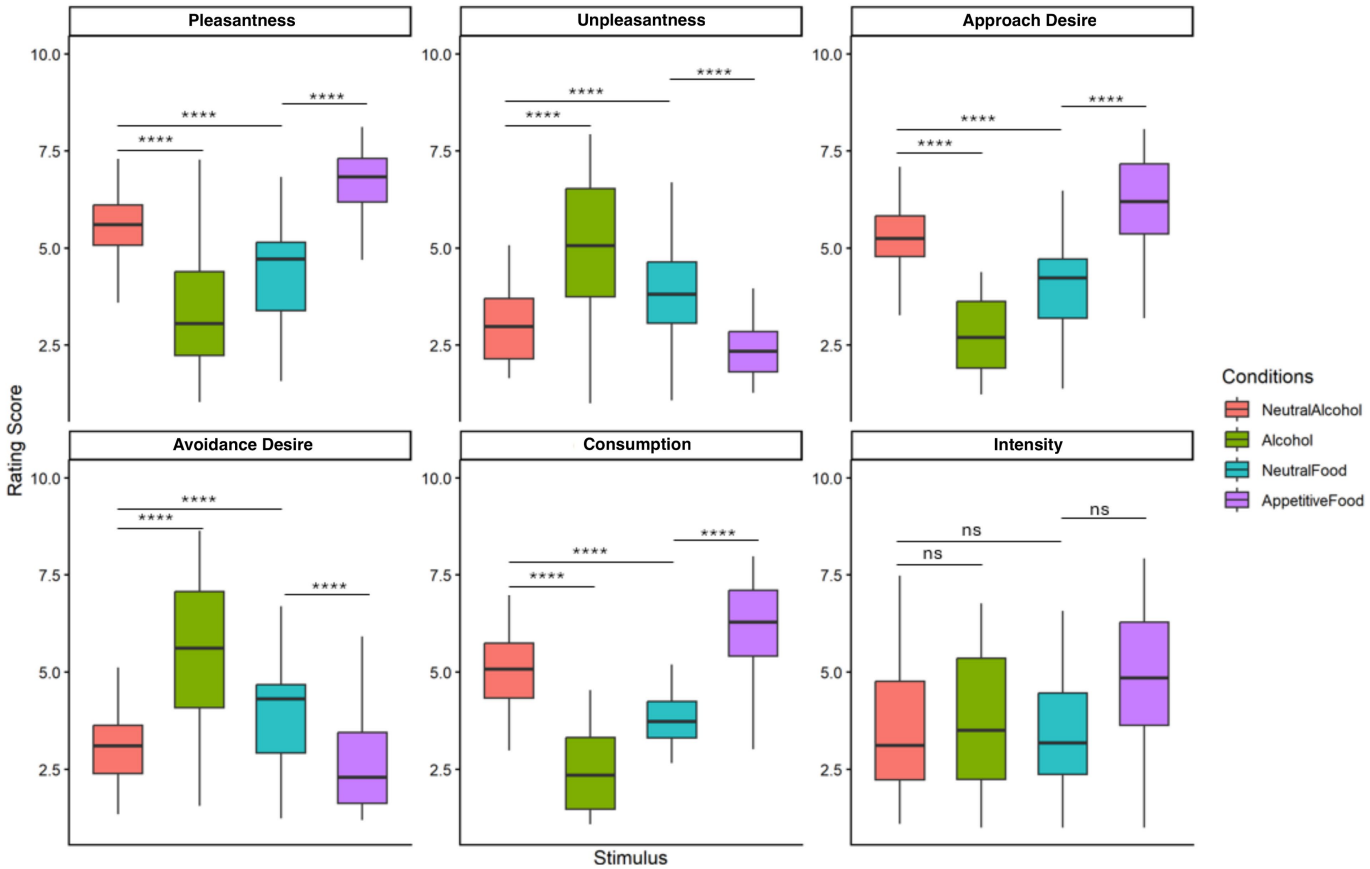
Rating scores across four stimulus conditions in the low AUDIT group for six emotional and motivational dimensions. Ratings were provided on a 9-point scale. Statistical differences were assessed using the Wilcoxon signed-rank test (ns = not significant, ^****^p < 0.0001).

Alcohol and food stimuli were significantly differently rated from their neutral counterparts on all dimensions (SOM, Figure 3, *p* < 0.0001), except for the Intensity dimension in the neutral alcohol–alcohol comparison. In the low-consumption group, alcohol and food stimuli were rated significantly differently from their neutral counterparts on all dimensions (*p* < 0.0001), except for intensity in the neutral alcohol–alcohol comparison (*p* = 0.89). The comparison between alcohol and food also showed highly significant differences across all dimensions, indicating distinct emotional and motivational responses. Notably, significant differences on the Avoidance dimension suggest that even low consumers showed clear approach-avoidance reactions to these stimuli.

#### 3.2.3 Higher-AUDIT scorers

**Figure 4:**
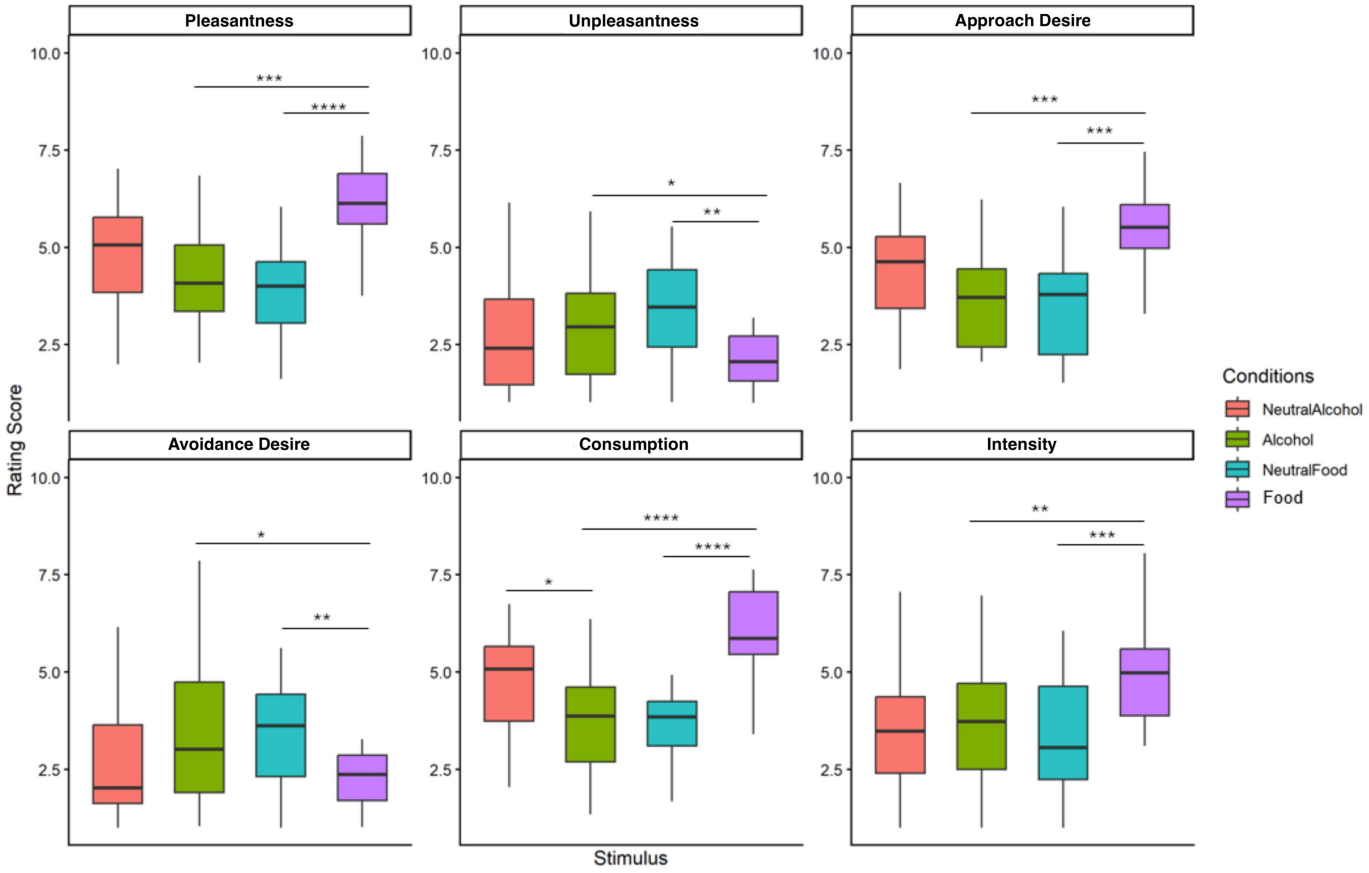
Rating scores across four stimulus conditions in the high AUDIT group for six emotional and motivational dimensions. Ratings were provided on a 9-point scale. Statistical differences were assessed using the Wilcoxon signed-rank test (ns = not significant, ^*^p < 0.05, ^**^p < 0.01, ^***^p < 0.001, ^****^p < 0.0001).

In the high-AUDIT group (see Figure 4), the comparison between alcohol and neutral alcohol stimuli revealed a significant difference only for the consumption dimension (*p* = 0.017), whereas no significant differences were observed for the other dimensions (*p* > 0.05). In contrast, comparisons between food and neutral food stimuli revealed significant differences across all dimensions, including pleasantness (*p* < 0.0001), unpleasantness (*p* = 0.001), approach desire (*p* < 0.001), Avoidance desire (*p* = 0.008), consumption desire (*p* < 0.0001), and intensity (*p* < 0.001). These findings suggest a stronger subjective differentiation between appetitive food stimuli and their neutral counterparts compared with alcohol stimuli in participants with higher AUDIT scores.

### 3.3 Neural responses

Because the study was exploratory regarding the spectral correlates of cue-reactivity, time–frequency analyses were performed separately for each canonical frequency band (delta, theta, alpha, beta). We acknowledge that this approach increases the number of statistical comparisons; however, the use of non-parametric cluster-based permutation tests strongly limits the risk of Type I error inflation by controlling for spatiotemporal dependencies in the data.

In addition, to address potential baseline differences between neutral stimulus categories, we directly compared neutral alcohol and neutral food conditions. This analysis was conducted to ensure that any effects observed in the main contrasts (alcohol vs. neutral; food vs. neutral) were not driven by intrinsic differences between the two neutral stimulus sets. The same preprocessing pipeline and non-parametric cluster-based permutation approach were applied. No significant clusters were observed between neutral alcohol and neutral food conditions across the examined frequency bands (all *p* > 0.05).

#### 3.3.1 Global average analyses

##### 3.3.1.1 Alcohol vs. neutral-alcohol

**Figure 5:**
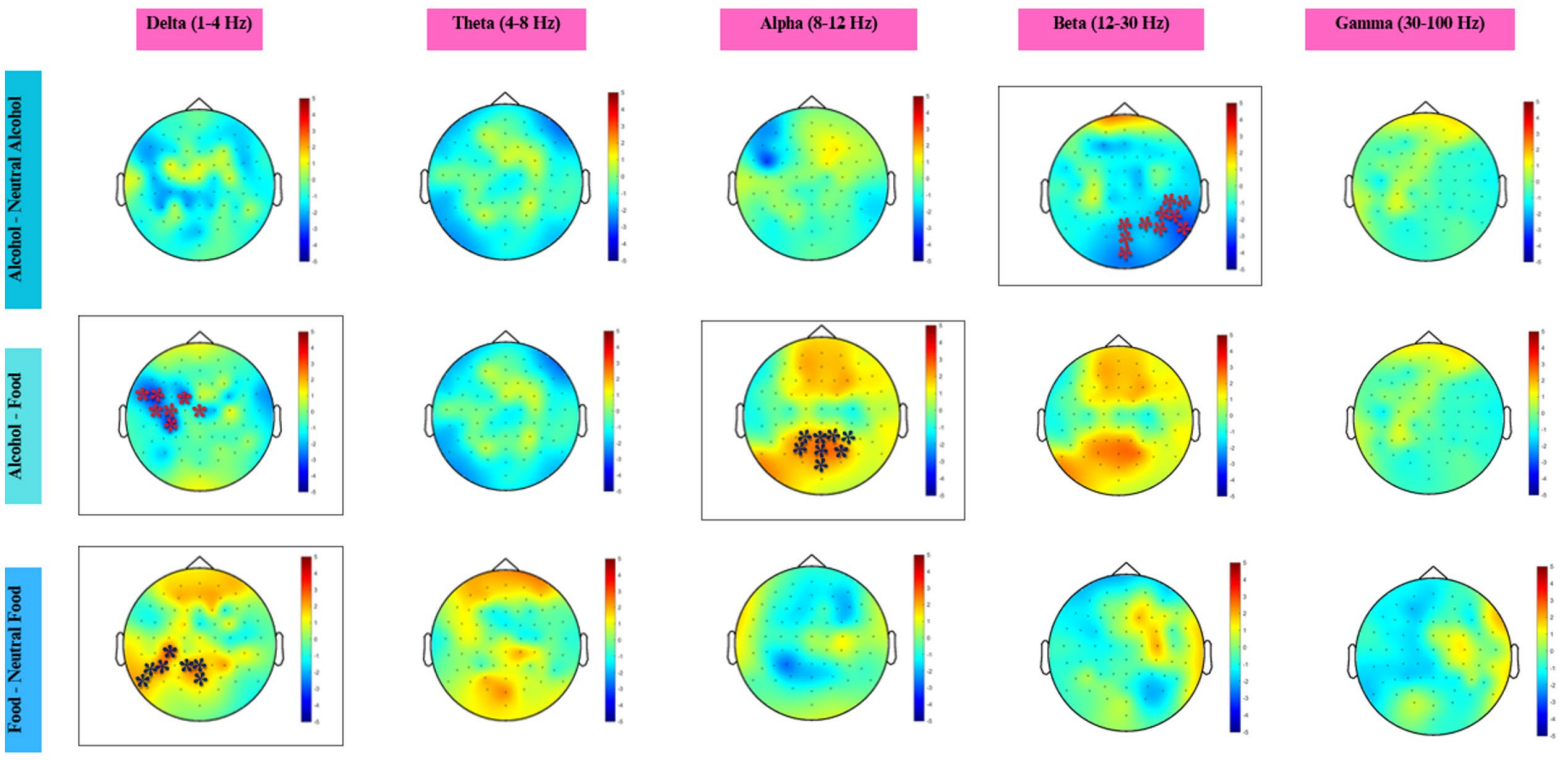
Topographical maps across five frequency bands for three condition contrasts. Colors represent statistical *t*-values obtained from the cluster-based permutation test. Red shades indicate positive clusters (higher power in the first condition), and blue shades indicate negative clusters (higher power in the second condition). Electrodes within significant clusters (*p* < 0.05) are marked with an asterisk (*).

In the beta band, a significant negative cluster was identified (cluster-level *p* = 0.014) over a broad right posterior region including Iz, Oz, POz, TP8, CP6, P6, P8, P10, PO8, and PO4 electrodes. The cluster-level effect size was small (Cohen’s *d* ≈ 0.13) (see Figure 5).

##### 3.3.1.2 Alcohol vs. food

In the delta band, a significant negative cluster was identified over left frontocentral and central regions (cluster-level *p* = 0.024), including FT7, FC5, FC1, C3, C5, CP3, and Cz electrodes. The cluster-level effect size was small (Cohen’s *d* ≈ 0.12), indicating a modest reduction in delta power in the food condition relative to the alcohol condition.

In the alpha band, a significant positive cluster was observed over posterior regions (cluster-level *p* = 0.031), encompassing P1, P3, Oz, POz, Pz, P2, PO3, and PO4 electrodes. The effect size was small (Cohen’s *d* ≈ 0.12).

##### 3.3.1.3 Food vs. neutral-food

In the delta band, a significant positive cluster was identified over posterior and centro-parietal regions (cluster-level *p* = 0.0316), including CP3, P1, P5, P7, P9, POz, and Pz electrodes. The cluster-level effect size was negligible (Cohen’s *d* ≈ −0.03). No statistically significant clusters were observed in the theta, alpha, beta, or gamma bands.

Across contrasts, statistically significant effects were limited. The alcohol vs. neutral-alcohol comparison revealed a significant beta-band cluster over right posterior regions. The alcohol vs. food contrast showed a significant decrease in delta activity over left frontocentral regions and an increase in posterior alpha activity. The food vs. neutral-food contrast revealed a significant delta-band cluster over posterior regions. Effect sizes across contrasts were generally small.

##### 3.3.1.4 Psychometric modulation of neural reactivity

To examine whether individual differences modulated cue-related neural reactivity, correlation analyses were conducted between condition-specific reactivity indices and questionnaire measures.

For the alcohol condition, beta-band reactivity (alcohol − neutral alcohol) showed a moderate positive association with depressive symptoms (BDI; *r* = 0.32, *p* = 0.025). However, this effect did not survive false discovery rate (FDR) correction across psychometric measures. No significant associations were observed between alcohol-related reactivity and AUDIT, STAI, or DEBQ subscales.

For the food condition, theta-band reactivity (food − neutral food) was negatively associated with restrained eating (DEBQ; *r* = −0.37, *p* = 0.011). This association remained significant after controlling for depressive and anxiety symptoms (partial *r* = −0.37, *p* = 0.011). No other significant associations were detected.

#### 3.3.2 Global average analysis in alcohol users

**Figure 6:**
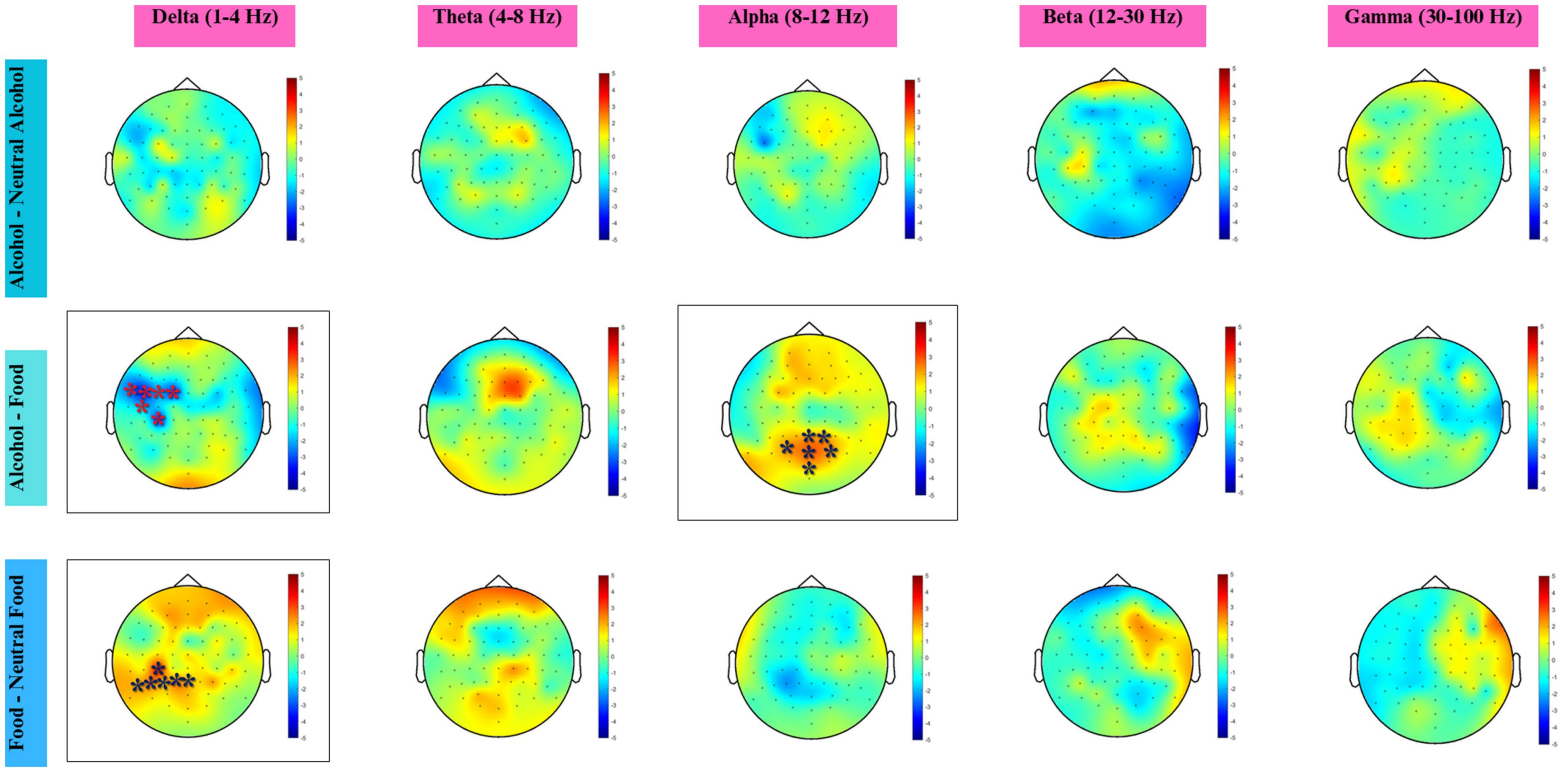
Topographical maps across five frequency bands for three condition contrasts. Colors represent statistical *t*-values obtained from the cluster-based permutation test. Red shades indicate positive clusters (higher power in the first condition), and blue shades indicate negative clusters (higher power in the second condition). Electrodes within significant clusters (*p* < 0.05) are marked with an asterisk (*).

Here, participants who had no experience with alcohol (AUDIT = 0) were excluded and only the EEG responses of individuals who used alcohol (38) were evaluated (see Figure 6).

##### 3.3.2.1 Alcohol vs. neutral-alcohol

No cluster reached significance in this constrast.

##### 3.3.2.2 Alcohol vs. food

In the delta band, a significant negative cluster was identified over the left frontocentral region (cluster-level *p* = 0.0238), including FT7, FC5, FC3, FC1, C5, and CP3 electrodes. The cluster-level effect size was small (Cohen’s *d* ≈ 0.13).

In the alpha band, a significant positive cluster was observed over parieto-occipital regions (cluster-level *p* = 0.0464), encompassing PO3, Oz, POz, Pz, P2, and PO4 electrodes. The effect size was small (Cohen’s *d* ≈ 0.06).

No statistically significant clusters were detected in the theta, beta, or gamma bands.

##### 3.3.2.3 Food vs. neutral-food

In the delta band, a significant positive cluster was identified over centro-parietal regions (cluster-level *p* = 0.0442), including CP3, P1, P3, Pz, P5, and P7 electrodes. The cluster-level effect size was small (Cohen’s *d* ≈ −0.07). No statistically significant clusters were observed in the theta, alpha, beta, or gamma bands.

#### 3.3.3 Group-level analysis based on lower and higher-AUDIT scorers

##### 3.3.3.1 Lower-AUDIT scorers group

**Figure 7:**
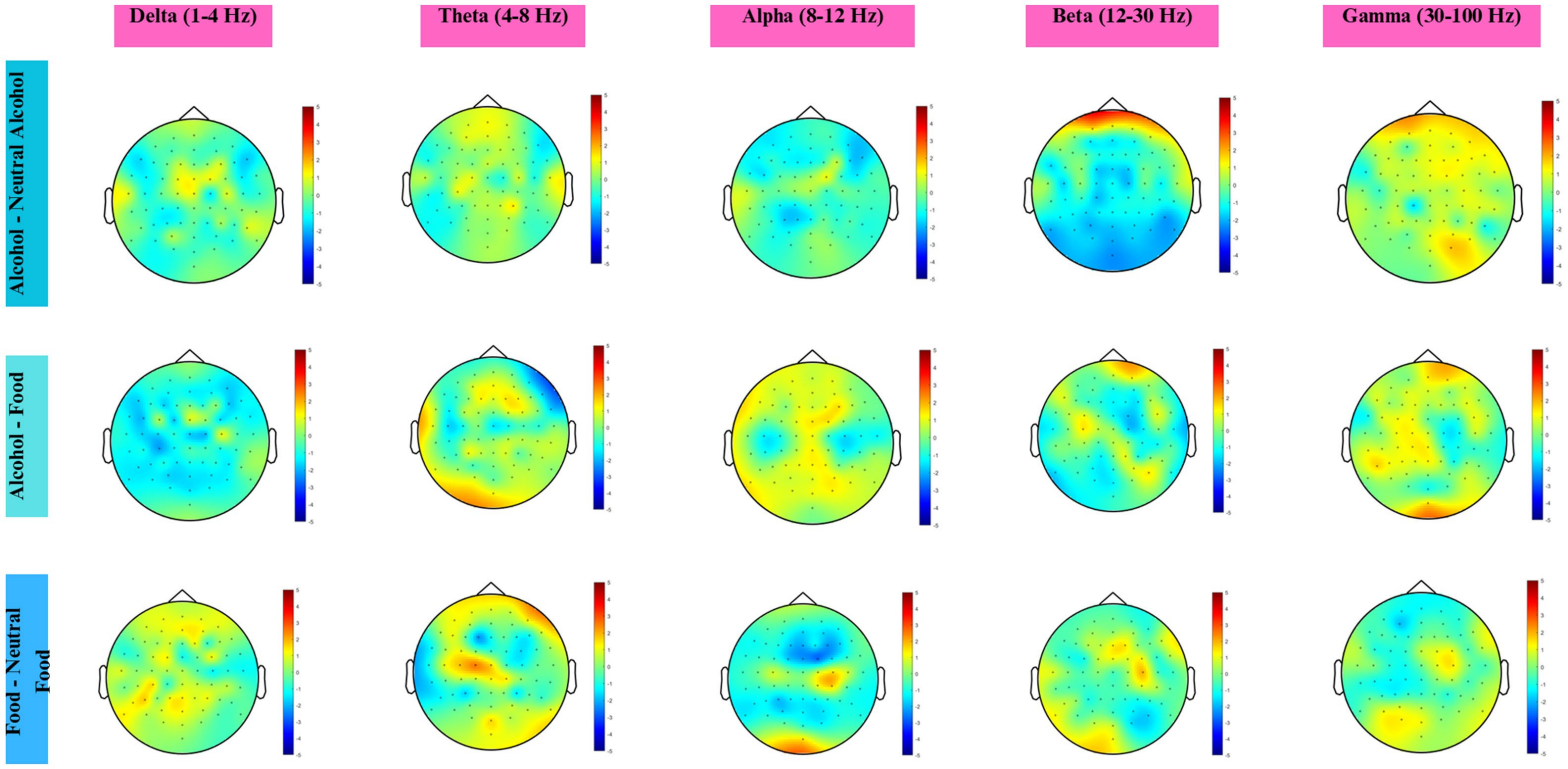
Topographic maps across five frequency bands for three condition contrasts, based on lower-AUDIT scorers (AUDIT ≤ 3). Colors represent statistical *t*-values obtained from the cluster-based permutation test. Red shades indicate positive clusters (higher power in the first condition), and blue shades indicate negative clusters (higher power in the second condition). Electrodes within significant clusters (*p* < 0.05) are marked with an asterisk (*).

No statistically significant clusters were found in any contrast (Figure 7).

##### 3.3.3.2 Higher-AUDIT scorers group

**Figure 8:**
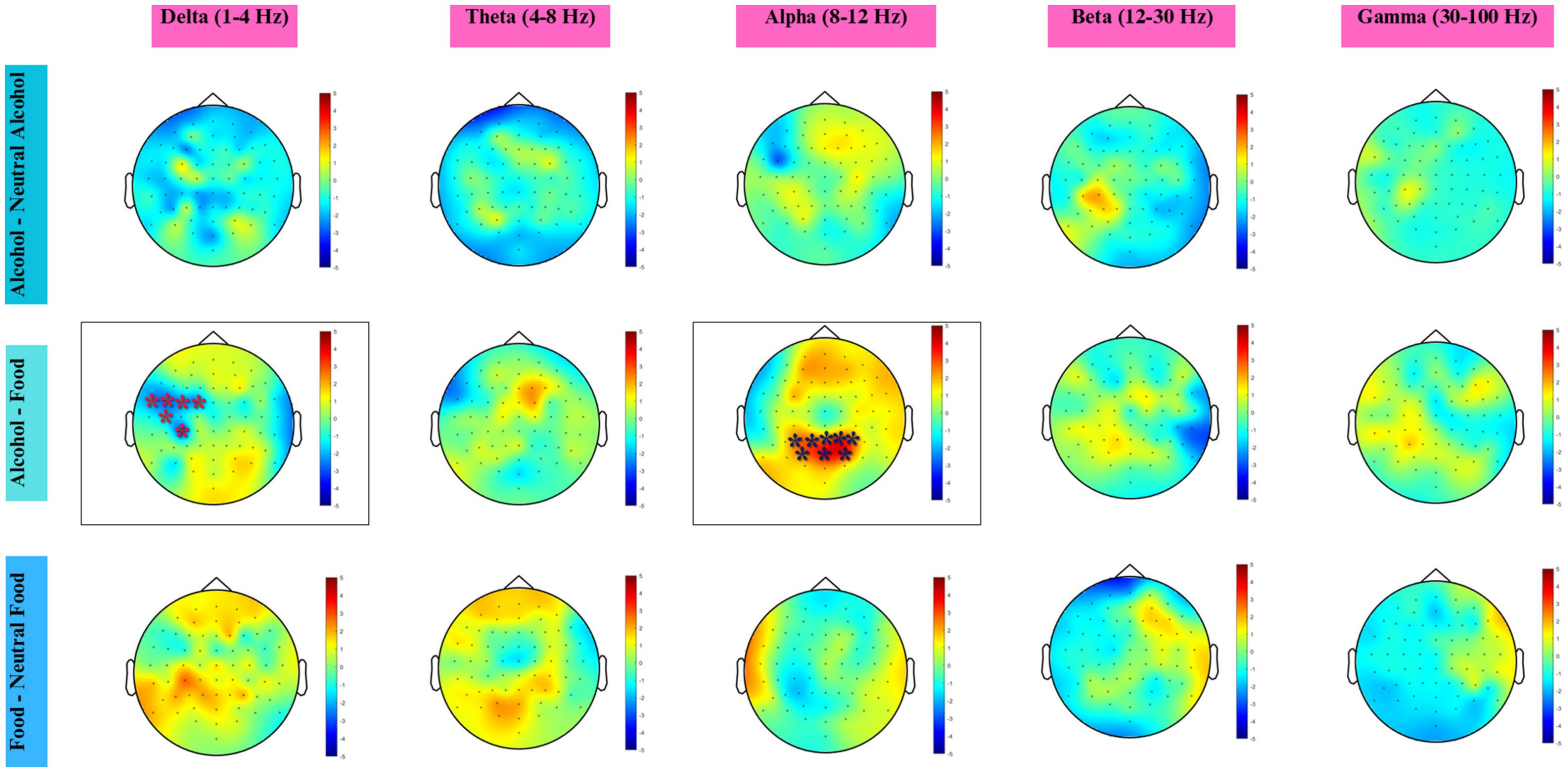
Topographic maps across five frequency bands for three condition contrasts, based on higher-AUDIT scorers (AUDIT > 3). Colors represent statistical *t*-values obtained from the cluster-based permutation test. Red shades indicate positive clusters (higher power in the first condition), and blue shades indicate negative clusters (higher power in the second condition). Electrodes within significant clusters (*p* < 0.05) are marked with an asterisk (*).

The corresponding results are shown in Figure 8. *Alcohol vs neutral-alcohol*. No statistically significant difference was found.

*Alcohol vs*. *food*. Significant cluster-level differences were detected, particularly in the delta and alpha bands. In the delta band, a negative cluster (cluster-level *p* = 0.022) was observed over the left frontocentral and centroparietal regions, including FT7, FC5, FC3, FC1, C5, and CP3 electrodes. The cluster-level effect size was small to moderate (Cohen’s d ≈ 0.18).

In the alpha band, a positive cluster (cluster-level *p* = 0.020) was identified over parieto-occipital regions, encompassing P1, P3, PO3, POz, Pz, P2, P4, and PO4 electrodes. The effect size was small (Cohen’s *d* ≈ 0.04).

No statistically significant clusters were detected in the theta, beta, or gamma bands.

*Food vs. neutral-food*. No statistically significant difference was found.

## 4 Discussion

The present study investigated the neural dynamics underpinning visual processing of food- and alcohol-related stimuli using EEG spectral analyses. Across participants, alcohol and food images elicited dissociable oscillatory patterns, particularly in the delta and alpha frequency bands, and these effects were modulated by individual differences in alcohol use.

### 4.1 Food vs. alcohol stimuli: motivational and embodied distinctions

Direct comparisons between alcohol and food cues revealed a robust dissociation: alcohol images induced decreased delta power over left fronto-central and centro-parietal regions, whereas food images elicited increased delta power over posterior regions. Delta oscillations have been broadly associated with motivational and internal attention processes (Knyazev, 2007, 2012) as well as bodily homeostasis and interoceptive dynamics (Marshall et al., 2002; Harmony, 2013). In the present study, the increase in delta for food cues aligns with the high intrinsic biological salience of food and its connection to gustatory-somatosensory representations (Carbine et al., 2018; Stockburger et al., 2009b; Wallroth et al., 2018). This strong neural response to food cues is consistent with the behavioral ratings observed in the present study. As illustrated in Figure 2, appetitive food stimuli elicited the highest scores across several motivational dimensions, including Pleasantness, Approach Desire, and Consumption Desire, clearly differentiating them from both alcohol and neutral stimuli. This pattern suggests that appetitive food images represented particularly salient motivational cues for participants in the present sample. One possible explanation lies in the distinction between primary and learned rewards. Food constitutes a biologically primary reinforcer directly linked to survival, whereas alcohol cues typically acquire their motivational value through learning and repeated consumption experiences. Consequently, in non-clinical populations, food-related cues may evoke stronger and more consistent motivational responses than alcohol-related cues. This interpretation is consistent with previous studies showing that appetitive food stimuli strongly capture attention and elicit robust behavioral and neural responses even in healthy individuals (Hung et al., 2020; Liu et al., 2022). More recent work further suggests that appetitive food cues may interact with inhibitory control mechanisms and action preparation systems, highlighting the complex interplay between motivational salience and regulatory processes during food cue processing (Bianco et al., 2023).

Turning to alcohol cues, the relative decrease in delta power observed in the present study may reflect attenuated internal motivational engagement toward alcohol compared with food, consistent with the idea that alcohol rewards rely more on learned associations than on innate homeostatic drivers (Versace et al., 2015; Kim et al., 2023). Alcohol-related cues have been widely studied in the framework of cue-reactivity and addiction neuroscience. Numerous studies have shown that alcohol-related stimuli can trigger enhanced attentional and neural responses, particularly in individuals with higher levels of alcohol consumption or alcohol dependence (Field and Cox, 2008; Heinz et al., 2009). More recent meta-analytic evidence confirms that alcohol cues reliably activate reward-related brain regions and attentional networks involved in motivational processing (Zeng et al., 2021). Within incentive sensitization models, repeated alcohol exposure progressively increases the motivational salience of alcohol-related cues, leading to stronger cue-reactivity responses in vulnerable individuals. The comparatively weaker delta response observed in the present non-clinical sample may therefore reflect the lower incentive salience of alcohol cues in individuals without problematic consumption patterns.

This interpretation is consistent with the observation that food images depicted solid stimuli with mastication-related affordances, whereas alcohol cues represented liquid ingestion. Food stimuli may therefore activate richer embodied representations than alcohol cues. Future studies including non-alcohol ingestible liquids and non-ingestive appetitive rewards will be necessary to determine whether the observed dissociation reflects ingestion modality or salience type. Individual differences in alcohol consumption may also influence cue-related motivational processing. Previous research has shown that individuals with higher levels of alcohol use often display enhanced attentional and neural responses to alcohol-related cues, reflecting increased incentive salience associated with repeated substance exposure (Field and Cox, 2008; Heinz et al., 2009). Although the present sample consisted primarily of non-clinical participants, the subgroup analyses based on AUDIT scores suggest that motivational responses to alcohol cues may already vary as a function of consumption habits. This observation is consistent with models proposing that repeated alcohol exposure progressively strengthens the motivational relevance of alcohol-related stimuli.

### 4.2 Spectral dynamics of alcohol cue processing in the broader reward literature

Increased alpha power over parieto-occipital regions during alcohol cue viewing converges with the well-established role of alpha oscillations in attentional inhibition and gating mechanisms (Foxe and Snyder, 2011; Jensen and Mazaheri, 2010; Klimesch, 2012). More recent work has further emphasized the role of alpha activity in coordinating large-scale brain networks involved in cognitive control and attention regulation (Sadaghiani and Kleinschmidt, 2016). Within this framework, the modulation of alpha power observed in the present study may reflect regulatory mechanisms involved in controlling attentional responses toward motivationally salient cues. Similar alpha effects are reported in time–frequency analyses of reward anticipation during tasks such as the Monetary Incentive Delay and Doors tasks (Dominguez-Centeno et al., 2020; Crane et al., 2023). The present findings may therefore indicate that alcohol cues elicit a top-down regulatory mode, particularly in individuals with greater alcohol involvement.

In addition to alpha effects, a decrease in beta power for alcohol images, mostly over posterior regions, was also observed. Beta-band oscillations have also been linked to inhibitory control and top–down regulatory mechanisms. In cognitive control paradigms, beta activity is frequently associated with the maintenance of the current cognitive state and with the suppression of inappropriate or premature responses (Engel and Fries, 2010). Experimental studies further suggest that beta oscillations play a key role in motor inhibition and response suppression processes, particularly during tasks requiring the inhibition of prepotent actions (Picazio et al., 2014). Interpreting the present findings within this framework suggests that the beta-band modulations observed during cue exposure may reflect neural processes involved in regulating approach tendencies toward motivational stimuli. Although our passive viewing paradigm does not allow mechanistic inference, this pattern resonates with reports of beta desynchronization during alcohol cue exposure in inhibitory contexts (Blanco-Ramos et al., 2022) and may reflect reduced cognitive control demands in the absence of overt action requirements. Theta and gamma bands did not show robust modulation in the present study. This absence of clear effects may be consistent with the passive viewing nature of the paradigm, as theta oscillations are often associated with cognitive control and conflict monitoring, whereas gamma activity is frequently linked to perceptual binding and active reward processing during tasks involving decision-making or reward anticipation.

Taken together, the delta, alpha, and beta patterns observed in the present study suggest that different oscillatory mechanisms may contribute to the processing and regulation of motivational cues, reflecting the interplay between salience detection, attentional control, and inhibitory regulation.

### 4.3 Influence of individual differences in alcohol use

Higher-AUDIT scorers exhibited the most pronounced differences between alcohol and food cues, characterized by decreased delta and increased alpha power in response to alcohol stimuli. Such oscillatory patterns are compatible with theoretical frameworks of cue reactivity and neural sensitization associated with repeated alcohol exposure (Versace et al., 2015; Kim et al., 2023), but several non-mutually exclusive mechanisms should be considered. Decreased delta activity has been linked to alterations in motivational engagement and salience processing, whereas increased alpha power has been associated with inhibitory control, attentional disengagement, or regulatory responses to salient cues.

In contrast, lower-AUDIT scorers showed no systematic oscillatory modulation, suggesting that alcohol cues elicited limited motivational engagement in this group. Importantly, these interpretations remain tentative given the exploratory nature of the spectral analyses and the absence of direct measures of physiological or motivational state (e.g., hunger, satiety, or craving). Overall, the present findings suggest that oscillatory signatures of cue reactivity may vary gradually across the continuum of alcohol use, rather than reflecting a categorical distinction restricted to clinically dependent profiles.

### 4.4 Limitations and perspectives

Several limitations should be acknowledged. First, alcohol use was assessed dimensionally in a non-clinical sample; AUDIT-based subgroups were exploratory and should not be interpreted clinically. In addition, eating behavior questionnaires were included primarily to characterize the sample and to explore dimensional associations with cue-related neural reactivity, rather than to define categorical subgroups. Accordingly, we did not stratify participants according to eating behavior profiles, as establishing meaningful subgroups based on DEBQ dimensions would require a study specifically designed and powered for that purpose. In the present dataset, DEBQ scores were examined at the correlational level, which revealed a significant association between restrained eating and food-related theta reactivity, but no broader pattern justifying subgroup-based interpretations. Future studies specifically targeting eating behavior phenotypes should determine whether distinct oscillatory responses to food cues emerge across restrained, emotional, or external eating profiles. Consistently, depression and anxiety scores remained in the non-clinical range and did not influence behavioral or neural measures, in line with a companion report on the same cohort (Zitouni et al., 2025). Second, each frequency band was analysed separately; although cluster-based permutation tests reduce spatiotemporal false positives, multiband inference was not possible. Third, the block-wise structure of the experimental paradigm may have introduced carryover or contextual effects across successive trials. Although a fully randomized event-related design could reduce such effects, the block structure was selected to ensure sufficient temporal stability for capturing postural adjustments and sustained neural dynamics during prolonged stimulus exposure. Importantly, block order was counterbalanced across participants and rest periods were included between blocks to mitigate fatigue and habituation. Fourth, the present design does not dissociate reward type, ingestion modality, and valence/arousal contributions; future studies incorporating non-alcohol liquids and non-ingestive rewards would be informative. Fifth, the design prioritised ecological passive viewing, preventing systematic examination of brain–behavior associations. Finally, posturographic data collected simultaneously are presented elsewhere (Duman et al., 2025).

## 5 Conclusion

Taken together, the findings indicate that food and alcohol cues elicit dissociable oscillatory signatures during passive viewing. Food cues preferentially engage delta oscillations linked to somatosensory and homeostatic salience, whereas alcohol cues elicit alpha increases and delta decreases consistent with attentional gating and motivational ambivalence. These patterns are most pronounced in individuals with higher alcohol use, suggesting that spectral signatures may serve as markers of motivational biases and their inter-individual variability.

## Data Availability

The raw data supporting the conclusions of this article will be made available by the authors, without undue reservation.
