# Supplementary material for: Brain signature of food and alcohol stimuli processing: a comparative EEG study
Source: Front Hum Neurosci. 2026 May 15;20:1748993. doi: 10.3389/fnhum.2026.1748993 (PMC13219378; doi:10.3389/fnhum.2026.1748993)
Supplement: Supplementary file 1 [file Table_1.docx]

| **Participant** | **Rejected Trials** | **Exclusion Criterion** |
| --- | --- | --- |
| P01 | 33 | > mean + SD |
| P24 | 35 | > mean + SD |
| P27 | 35 | > mean + SD |
| P28 | 33 | > mean + SD |
| P31 | 35 | > mean + SD |
| P37 | 40 | > mean + SD |
| P51 | 36 | > mean + SD |
| P53 | 36 | > mean + SD |
| P57 | 34 | > mean + SD |

Table S1. Number of rejected trials for each participant after EEG preprocessing. The exclusion threshold was defined as one standard deviation above the mean number of rejected trials across participants (mean = 25.07, SD = 7.34, threshold = 32.41). Participants exceeding this threshold were excluded from further analyses.
